# High-Accuracy, Compact Scanning Method and Circuit for Resistive Sensor Arrays

**DOI:** 10.3390/s16020155

**Published:** 2016-01-26

**Authors:** Jong-Seok Kim, Dae-Yong Kwon, Byong-Deok Choi

**Affiliations:** Department of Electronic Engineering, Hanyang University, 222 Wangsimni-ro, Deongdong-gu, Seoul 04763, Korea; jskim383@hanyang.ac.kr (J.-S.K.); dy.kwon87@gmail.com (D.-Y.K.)

**Keywords:** resistive sensor, sensor array, crosstalk, read-out circuit, row driver

## Abstract

The zero-potential scanning circuit is widely used as read-out circuit for resistive sensor arrays because it removes a well known problem: crosstalk current. The zero-potential scanning circuit can be divided into two groups based on type of row drivers. One type is a row driver using digital buffers. It can be easily implemented because of its simple structure, but we found that it can cause a large read-out error which originates from on-resistance of the digital buffers used in the row driver. The other type is a row driver composed of operational amplifiers. It, very accurately, reads the sensor resistance, but it uses a large number of operational amplifiers to drive rows of the sensor array; therefore, it severely increases the power consumption, cost, and system complexity. To resolve the inaccuracy or high complexity problems founded in those previous circuits, we propose a new row driver which uses only one operational amplifier to drive all rows of a sensor array with high accuracy. The measurement results with the proposed circuit to drive a 4 × 4 resistor array show that the maximum error is only 0.1% which is remarkably reduced from 30.7% of the previous counterpart.

## 1. Introduction

**Figure 1:**
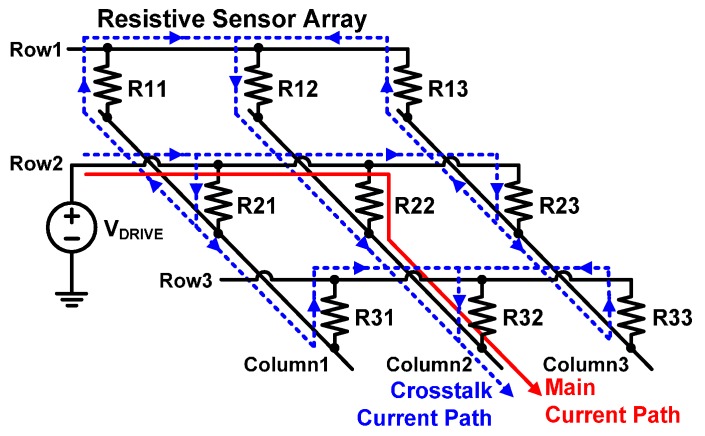
Crosstalk current paths in a resistive sensor array.

Resistive sensors, such as piezoresistive tactile sensors [1,2,3,4,5,6], infrared sensors [7,8,9,10], and light-dependent resistors [11,12], have been widely used for measurement or instrumentation in a variety of fields. These sensors are usually applied in the form of an array to obtain a required measurement resolution. When we use resistive sensor arrays, we should consider the crosstalk current between each sensor element in the array [13,14,15,16]. The crosstalk currents can flow through the unintended current paths when we read the voltage of the resistive sensor element of interest in a selected row of the array. For example, as shown in Figure 1, let us assume that we read the resistance of R22 by applying the driving voltage (V_DRIVE_) to the second row and measuring the voltage at Column 2 terminal. Then, other than the current flowing through R22, the unintended currents are added through the dotted path to Column 2 terminal, which pass through all the other resistor elements (R11, R12, R13, R21, R23, R31, R32, and R33 in Figure 1). Therefore, we cannot find a true value of R22 by measuring the voltage at the column terminal. This causes the accuracy problem in the sensor system.

**Figure 2:**
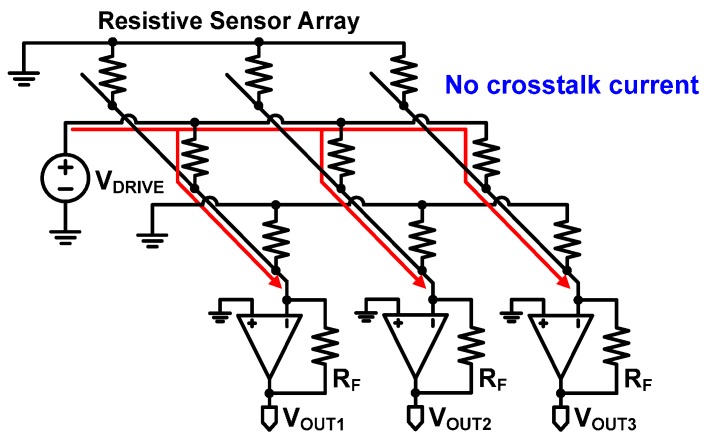
Zero-potential scanning method to eliminate the crosstalk current.

The crosstalk current problem in the sensor array has been addressed in the previous literature, and several solutions have been proposed. The crosstalk current can be avoided by inserting a diode [17,18] or a transistor [19,20] for each element in the sensor array, but these methods are seldom used for integrated sensors in VLSI/MEMS chips since the system complexity is drastically increased due to the additional diodes, transistors, and physical wires. Voltage feedback methods have been proposed to remove the crosstalk current [11,21,22], but it is known that those methods still suffer from the measurement errors which are increased as a sensor array becomes large [23]. The most widely used method to solve the crosstalk current problem is the zero-potential scanning method shown Figure 2 [13,14,15,16,21,24,25,26,27,28,29,30]. This method has an operational amplifier at each column terminal. Since both the negative and positive input voltages of the operational amplifier must be equal, due to the virtual short characteristics of an operational amplifier, all the columns and the rows have the same voltage of 0 V except for a selected row whose voltage is V_DRIVE_. Consequently, the currents only flow through the resistive sensor elements in the selected row without crosstalk currents.

The zero-potential scanning method can be classified into two groups based on type of a row driver: a row driver composed of digital buffers (or multiplexers) [13,15,16,24,25,26,27] (which is referred to as Type I in this paper) and a row driver composed of operational amplifiers [14,28,29,30] (which is referred to as Type II in this paper). Although both of the types were proposed to remove the crosstalk current, we found that Type I row driver still suffers from the inaccuracy problem and Type II row driver has the drawbacks of high power consumption, high cost, and high system complexity. These issues will be analyzed in detail in Section 2.

In this paper, we analyze and reveal the drawbacks of the previous circuits and propose a new zero-potential scanning method with a compact row driver that alleviates the drawbacks of the Type II row driver while maintaining the advantages. The paper is organized as follows. In Section 2, we analyze the previous circuits to reveal their limitations. The structure and operation principle of the proposed circuit are described in Section 3. In Section 4, the design, simulation, and measurement results are discussed, and performance comparisons are presented. Finally, conclusions are given in Section 5.

## 2. Analysis of Previous Row Drivers for Resistive Sensor Arrays

As described in Section 1, the zero-potential scanning method is widely used for resistive sensor arrays to eliminate the crosstalk current. There are two types of row drivers for the zero-potential scanning method: a row driver composed of digital buffers and a row driver composed of operational amplifiers. In this section, we analyze these two previous types from the perspective of the accuracy and the system complexity.

### 2.1. Row Driver Composed of Digital Buffers (Type I)

**Figure 3:**
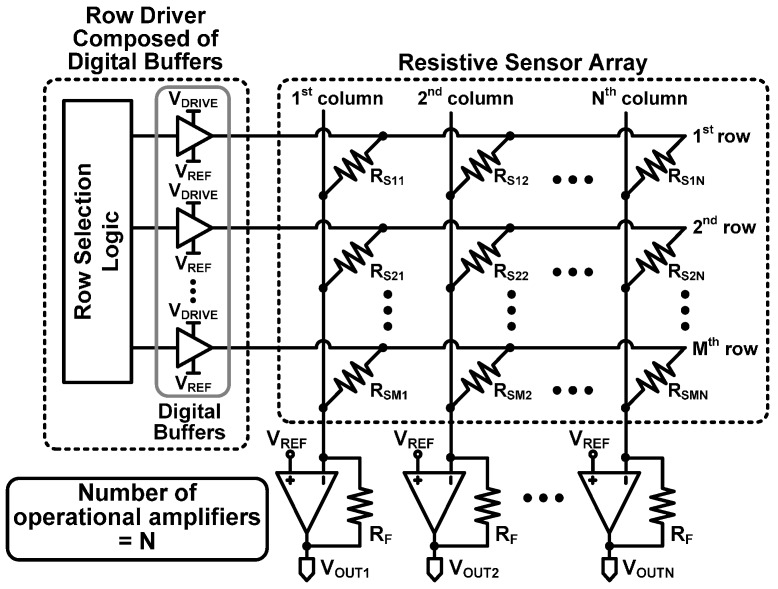
Zero-potential scanning method with a row driver composed of digital buffers (Type I).

The row driver composed of digital buffers in Figure 3, which is referred to as Type I here, has been proposed for resistive sensor arrays [13,15,16,21,24,25,26,27]. The system is made up of a resistive sensor array, column operational amplifiers, and a row driver including digital buffers.

**Figure 4:**
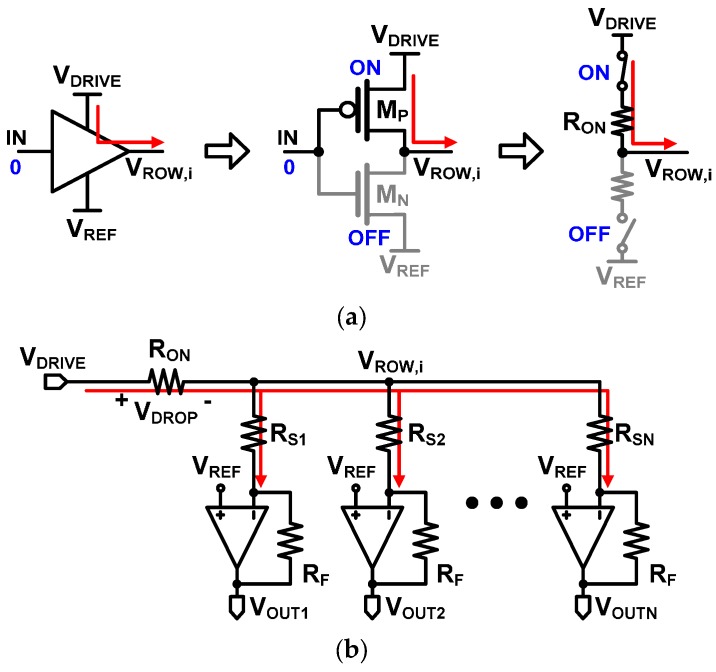
(**a**) Equivalent switch model of a digital buffer; and (**b**) equivalent circuit model of a resistive sensor array with the Type I row driver.

In order to verify how accurate row driver Type I is, we will calculate the output voltage of a column operational amplifier by using the equivalent circuit model of Type I shown in Figure 4. As shown in Figure 4a, a digital buffer is usually an inverter composed of PMOS (M_P_) and NMOS (M_N_) transistors. It can be modeled as a simple switch circuit which has some on-resistance (R_ON_). Therefore, when a digital buffer delivers V_DRIVE_ to a selected row, the Type I circuit can be modeled as shown in Figure 4b. When a digital buffer drives a selected row, a static current flows through R_ON_ thus creating a voltage drop (V_DROP_) across R_ON_. The voltage drop across R_ON_, V_DROP_, can be expresses as:

(1) $$V_{DROP}=\left(V_{DRIVE}-V_{REF}\right)\frac{R_{ON}}{R_{TOT}+R_{ON}}$$

where V_DRIVE_ is the row driving voltage, V_REF_ is the reference voltage tied to the positive inputs of all the column operational amplifiers, and R_TOT_ is the total resistance of all the resistive sensor elements in the selected row. Since they are connected in parallel, R_TOT_ is expressed as:

(2) $$\frac{1}{R_{TOT}}=\sum_{j=1}^{N}\frac{1}{R_{Sj}}$$

Using Equations (1) and (2), we can calculate the output voltage of the *jt*h column operational amplifier, V_OUT,j_ as:

(3) $$V_{OUT,j}=V_{REF}-\left(V_{ROW,i}-V_{REF}\right)\frac{R_{F}}{R_{Sj}}$$

(4) $$=V_{REF}-\left(V_{DRIVE}-V_{DROP}-V_{REF}\right)\frac{R_{F}}{R_{Sj}}$$

(5) $$=V_{REF}-\left(V_{DRIVE}-V_{REF}\right)\frac{R_{TOT}}{R_{TOT}+R_{ON}}\frac{R_{F}}{R_{Sj}}$$

(6) $$=V_{REF}-\left(V_{DRIVE}-V_{REF}\right)\frac{1}{1+R_{ON}/R_{TOT}}\frac{R_{F}}{R_{Sj}}$$

where V_ROW,i_ is the *i*th row voltage, R_Sj_ is the resistance of the sensor element at the intersection of the selected row and *j*th column.

Equation (6) clearly reveals that the *j*th column voltage, V_OUT,j_, is dependent upon the sensor elements of the other columns, because R_TOT_ is the total resistance of all the resistive sensor elements in the same row. If R_ON_ = 0, the equation is reduced to V_OUT,j_ = V_REF_ − (V_DRIVE_ − V_REF_) R_F_/R_Sj_ which shows that the column voltage is determined by the sensor element in the *j*th column alone independently of the other sensor elements in the same row. However, in real digital buffers, R_ON_ cannot be zero, because R_ON_ is the on-resistance of the transistor used in the digital buffer as shown in Figure 4a. We measured the on-resistances of several commercially available digital buffers and found that they are in the range of tens of ohms [31,32,33,34]. Therefore, in Equation (6), if R_ON_/R_TOT_ is not much smaller than 1, V_OUT,j_ is affected by R_ON_/R_TOT_. As expressed in Equation (2), R_TOT_ becomes smaller as the number of columns (N) increases and/or as R_Sj_ decreases. Conclusively, V_OUT,j_ of Type I is affected by the other sensor elements in the same row, and this causes inaccuracy in reading the sensor resistance. This inaccuracy problem with the Type I row driver will be also presented through simulations and measurements in Section 4.

### 2.2. Row Driver Composed of Operational Amplifiers (Type II)

**Figure 5:**
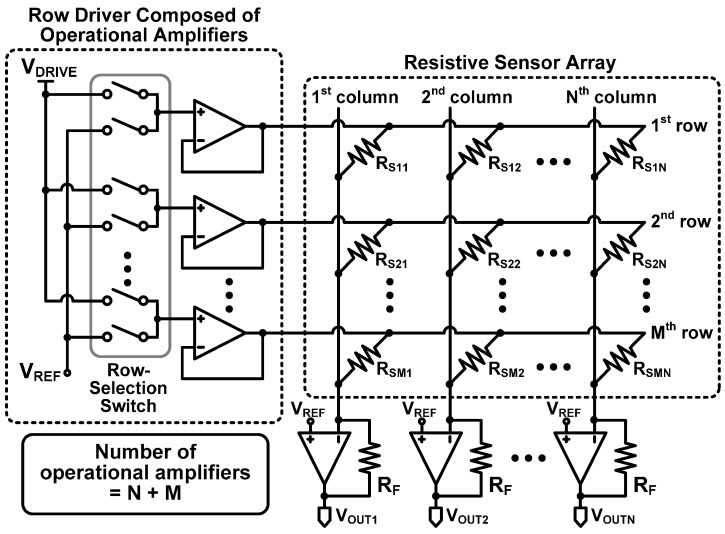
Zero-potential scanning method with a row driver composed of operational amplifiers (Type II).

Figure 5 shows the zero-potential scanning method using a row driver composed of operational amplifiers (referred to as Type II here) [14,28,29,30]. Both Type I and Type II commonly use operational amplifiers at the column terminals, but Type II also uses operational amplifiers instead of digital buffers used in the Type I row driver.

**Figure 6:**
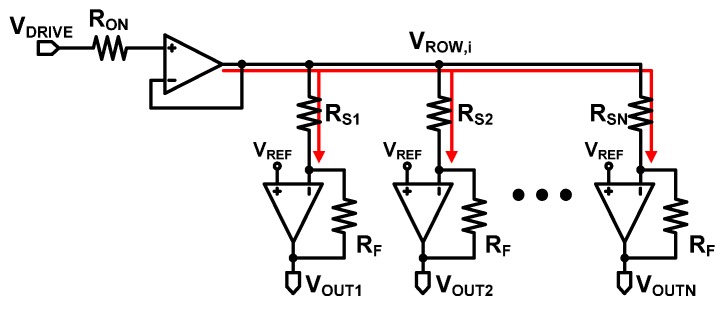
Equivalent circuit model of a resistive sensor array with row driver Type II.

Whereas Type I suffers from inaccuracy in reading a sensor resistance, as analyzed in Section 2.1, Type II is effective in achieving sufficient accuracy. Figure 6 shows the equivalent circuit model of Type II. The on-resistance of the selection switch in Figure 5 is represented as R_ON_. However, because no static current flows through R_ON_, no voltage drop occurs across it, the positive input of the operational amplifiers is V_DRIVE_, and the row voltage V_ROW,i_ is virtually equal to V_DRIVE_ due to the virtual short characteristic of the operational amplifiers. Consequently, the output voltage of the *j*th column amplifier of Type II can be expressed as:

(7) $$V_{OUT,j}=V_{REF}-\left(V_{DRIVE}-V_{REF}\right)\frac{R_{F}}{R_{Sj}}$$

In Equation (7), it is clear that the column voltage is determined by the resistive sensor element in the *j*th column alone independently of the other sensor elements in the same row. Therefore, the Type II row driver can avoid the inaccuracy problem, unlike row driver Type I.

However, this requires a large number of operational amplifiers. Since an operational amplifier consumes a considerable static power, it can significantly increase the power consumption, especially for a large sensor array. For example, a sensor array with N columns and M rows, the Type row driver II requires N + M operational amplifiers, while the Type I row driver uses M operational amplifiers. A large number of operational amplifiers also increases the system complexity, volume, and cost.

## 3. Proposed Row Driver for Zero-Potential Scanning Method (Type III)

**Figure 7:**
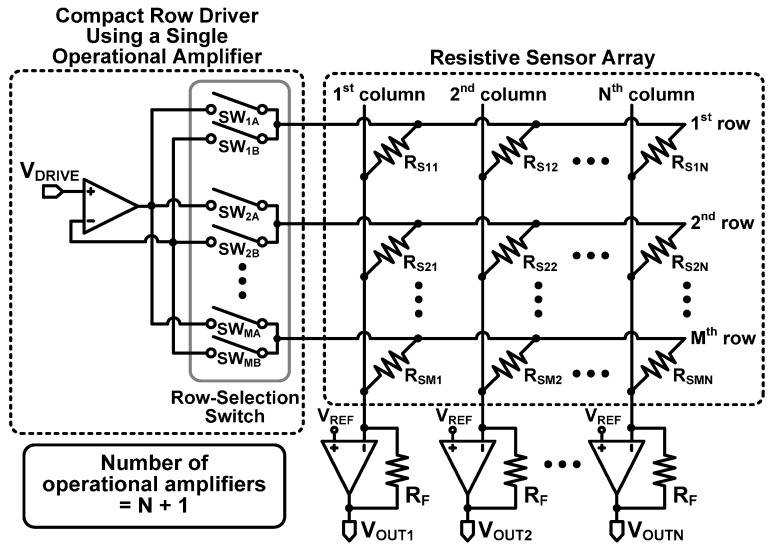
Proposed zero-potential scanning method with the proposed compact row driver using a single operational amplifier (Type III).

To resolve the problems of inaccuracy of Type I and high circuit complexity of Type II mentioned in Section 2, we propose a row driver using a single operational amplifier for the zero-potential scanning method (referred to as Type III) as shown in Figure 7. The Type II row driver uses an operational amplifier for each row, but the proposed row driver uses a single operational amplifier for the entire rows, while maintaining the merit of Type II.

**Figure 8:**
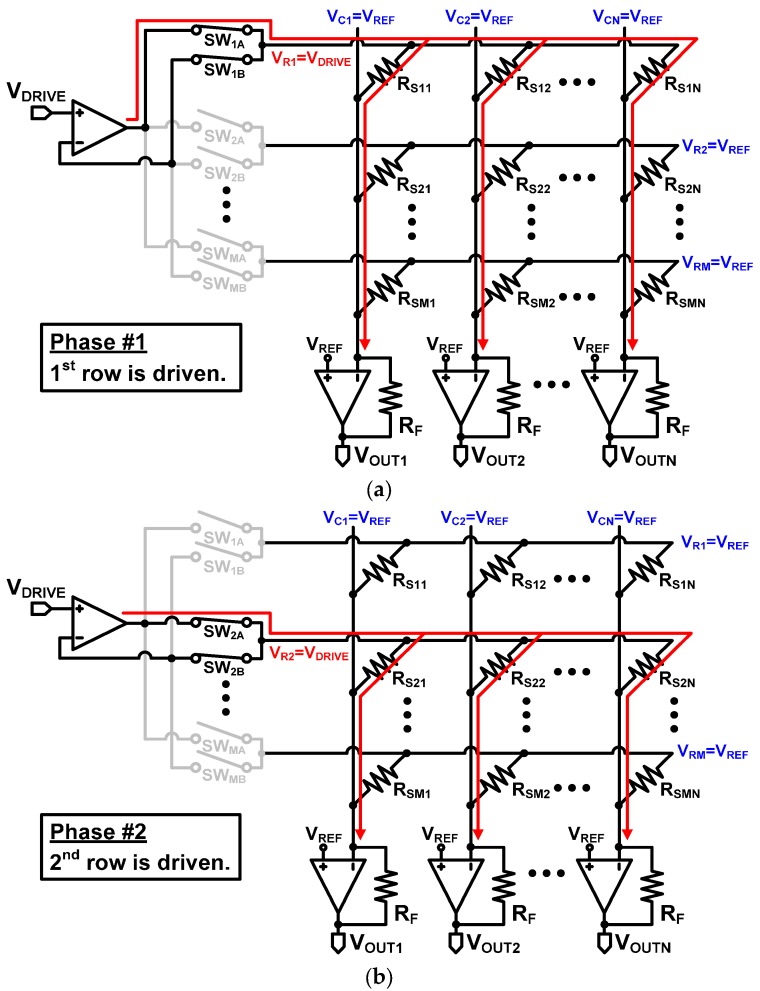
Scanning operation of the proposed row driver Type III. (**a**) 1st and (**b**) 2nd row is driven.

The scanning operation of the proposed circuit is illustrated in Figure 8. When the first row is driven, a pair of switches, SW_1A_ and SW_1B_, are turned on, while the other switches are turned off. Then, both the output and the negative input of the operational amplifier are connected to the first row. When the second row is driven, SW_2A_ and SW_2B_ are turned on, and the other switches are turned off. With this driving repeated throughout the remaining row, the proposed Type III can drive all the rows using a single operational amplifier.

**Figure 9:**
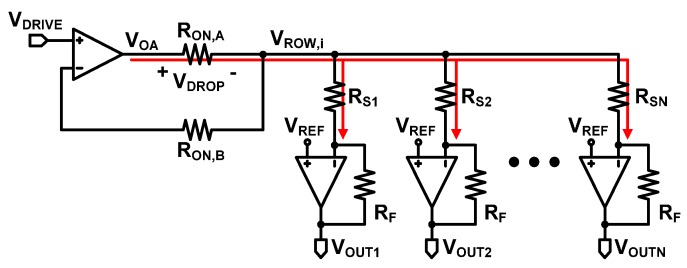
Equivalent circuit model of a resistive sensor array with the proposed Type III row driver.

When a selected row is driven by a row driver, it can be modeled by the equivalent circuit in Figure 9, where R_ON,A_ and R_ON,B_ represent the on-resistnace of the row selection switches SW_1A_ and SW_1B_ in Figure 8, respectively. As shown in the figure, the row driving current flows through R_ON,A_, but no current flows through R_ON,B_, because the input impedance of the operational amplifier is almost infinite. Consequently, notwithstanding the fact that the row driving current makes the voltage drop across R_ON,A_, the row voltage V_ROW,j_ is regulated to V_DRIVE_ by the virtual short characteristic of the operational amplifier. Therefore, the output voltage of the *j*th column operational amplifier of Type III can be expressed as:

(8) $$V_{OUT,j}=V_{REF}-\left(V_{DRIVE}-V_{REF}\right)\frac{R_{F}}{R_{Sj}}$$

This equation is exactly the same with Equation (7), and V_OUT,j_ is determined by the sensor element in the *j*th column alone and is independent of the other sensor elements; therefore, the proposed Type III driver is as accurate as the Type II in measuring the sensor resistance. Additionally, it should be noted that all the unselected rows are not electrically floating but also regulated to V_REF_ by the column drivers (not the row drivers). This means that no current flows through the sensor elements of the unselected rows. Consequently, the accuracy of the Type III row driver is expected to be identical to that of the Type II row driver, because no row or column is eletrcially floating in both of the row drivers. The simulation and measurement results will be presented in Section 4.

From the operation described above, we can find that the proposed Type III driver can drive all of the rows using a single operational amplifier without the inaccuracy problem. That is to say, the proposed Type III driver can drastically reduce the number of operational amplifiers. For example, to drive N × M resistive sensor array, the proposed Type III needs only N + 1 operational amplifiers, which is a markedly reduced number compared with that of Type II, which uses N + M operational amplifiers. With the reduced number of operational amplifiers, the proposed Type III can considerably reduce the power consumption, system complexity, and cost compared with Type II. Although each row has two additional switches, they are much simpler than the operational amplifier.

The characteristics of three types of row drivers are summarized in Table 1. All types of the row drivers can eliminate crosstalk current because they are all based on the zero-potential scanning method. However, Type I significantly degrades the accuracy because the voltage measurement of a sensor in a row is affected by the other sensors in the same row. We reveal that this inaccuarcy is not caused by the crosstalk current, but comes from the row driver circuit itself. Type II successfully eliminates both inaccuracy and crosstalk current, but leads to high power consumption, high cost, and high complexity, since it requires a large number of amplifiers. On the contrary, the proposed Type III not only removes both crosstalk and inaccuracy, but requires only one amplifier for all rows. Therefore, we can conclude that the proposed Type III simulataneously takes the advantages of both Type I and II.

**Table 1:** Comparison of three types of row drivers.

	Type I	Type II	Type III (Proposed)
Crosstalk current	eliminated	eliminated	eliminated
Inaccuracy	exists	eliminated	eliminated
Number of operational amplifiers (for N × M array)	N	N + M	N + 1

## 4. Results and Discussion

To compare the characteristics of the previous Type I, II, and the proposed Type III row driver circuits, before real implementation and measurement, we performed PSPICE simulations [35]. The resistance of the sensor elements are assumed to be in the range from 100 Ω to 1 MΩ, which can cover most of the previously reported resistive sensors [1,2,3,4,6,7,8,9,11,12,14].

For accurate read-out of the sensor resistance, we should be careful to choose an operational amplifier since an operational amplifier has non-ideal properties affecting the accuracy. First, an operational amplifier should provide a low-offset voltage which is a source of output voltage error. Second, the open-loop gain should be large enough to reduce gain error. Third, it should provide a sufficient current driving capability. In addition, an input bias current flowing into both inputs of an operational amplifier should be very small. Considering these requirements, we use the high precision amplifier, LMP7701 for both column and row amplifiers [36]. The operational amplifier has the specifications as follows: typical offset voltage = ±37 μV, open-loop gain = 121 dB, maximum driving current = 86 mA, and maximum input bias current = 1 pA. With these specifications, errors caused by the non-ideal properties of the operational amplifier can be neglected.

If inccuracy exists in reading a sensor resistance, it causes the output voltage to deviate from the true value as discussed in Section 2; thus, we can define the percentage error of the output voltage as:

(9) $$e\left(\%\right)=\left|\frac{V_{OUT}-V_{IDEAL}}{V_{IDEAL}}\right|\times 100$$

where V_OUT_ is measured (or simulated) the output voltage of the column amplifier and V_IDEAL_ is the ideal output voltage which should be obtained without any inaccuracy as expressed in Equations (7) or (8).

**Figure 10:**
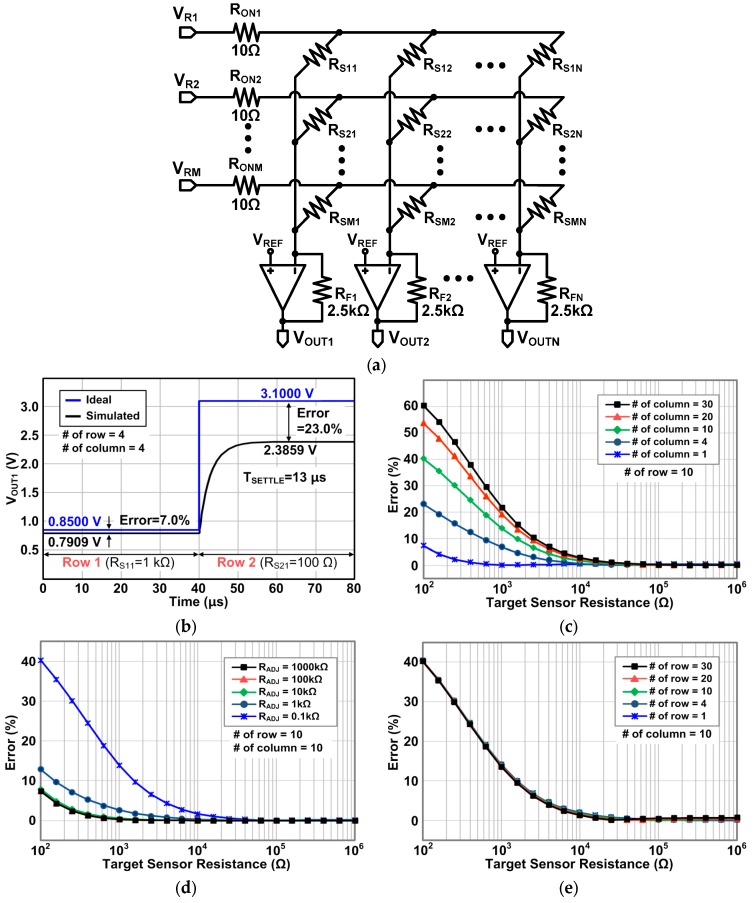
(**a**) Schematic for the simulations of the Type I row driver; (**b**) transient simulation results; (**c**) output errors as a function of the sensor resistance with different column numbers; (**d**) output errors as a function of the sensor resistance with different adjacent sensor resistance (R_ADJ_), and (**e**) output errors as a function of the sensor resistance with different row numbers.

Figure 10a shows the schematic for simulations of row driver Type I with N × M resistive sensor array. As described in Section 2, the digital buffer in row driver Type I can be modeled as a resistor, R_ON_. Here, R_ON_ is set at 10 Ω which is a typical resistance found in many commercial digital buffers [31,32,34]. Now we will observe the output voltage at Column 1 (V_OUT1_), where the resistor to be sensed, R_S11_ and R_S21_, are assumed to be 1 kΩ and 100 Ω, respectively. All of the other resistors are assumed to be 100 Ω. In this simulation, when a row is selected, the row is driven to V_DRIVE_ (0.5 V) and all the other rows are biased to V_REF_ (0.6 V).

The transient simulation results of row driver Type I are shown in Figure 10b. By selecting Row 1 and reading the output voltage at Column 1, we obtain the value of R_S11_ (assumed to be 1 kΩ here). V_OUT1_ should be 0.85 V if R_ON_ is ideally 0 Ω in Equation (6). However, as shown in the figure, the error of 7.0% is observed, which agrees very well with the error predicted in Equations (6) and (9). Then, we select Row 2 and read the output voltage at Column 1 to find out the value of R_S21_ (assumed to be 100 Ω here). Ideally, V_OUT1_ should be 3.1 V, but the output error reaches 23.0% as also calculated in Equations (6) and (9). The simulated 99% settling time is 13 μs.

To also find the impact of the number of columns, we performed the simulations by changing the number of columns while fixing the number of rows at ten. Figure 10c shows the output voltage error as the sensor resistance to be measured (which is referred to as target sensor here) is swept from 100 Ω to 1 MΩ, while all of the other sensor resistances in the same row stay at 100 Ω. If the target sensor resistance is 1 MΩ, which is 10,000 times larger than the other sensor elements, the error is very small. However, if the target sensor is 100 Ω, the error is drastically increased. If the target sensor is 100 Ω, the simulation result with a single column shows that the error is under 10%. However, with 10 columns, the error reaches almost 40%, and with 30 columns, the error is increased to over 60%. These results agree very well with the expectation from Equation (6). Figure 10d shows the output voltage error for different resitance of all the other sensor elements (R_ADJ_) in a 10 × 10 array. R_ADJ_ is swept from 100 Ω to 1000 kΩ. As expected in Equations (2) and (6), the error is increased as R_ADJ_ becomes similar to R_ON_ (100 Ω here). When R_ADJ_ is 1000 kΩ, the error is 7.3%, but when R_ADJ_ is decreased to 100 Ω, the error is increased to 40.2%. From the simulation results in Figure 10c,d, we can find that the Type I row driver suffers from the reading inaccuracy when the array becomes larger and R_ADJ_ becomes similar to R_ON_; therefore, it is not suitable for large sensor array applications.

Figure 10e shows the output voltage error for different numbers of rows with ten columns. There is no difference in the error for various numbes of rows, because the error is caused by R_ON_ and the currents flows through R_ON_ of the selected row only. In other words, all the other rows, except the selected row, has no current, because their row and column voltages are tied to V_REF_ as explained in Section 2.

**Figure 11:**
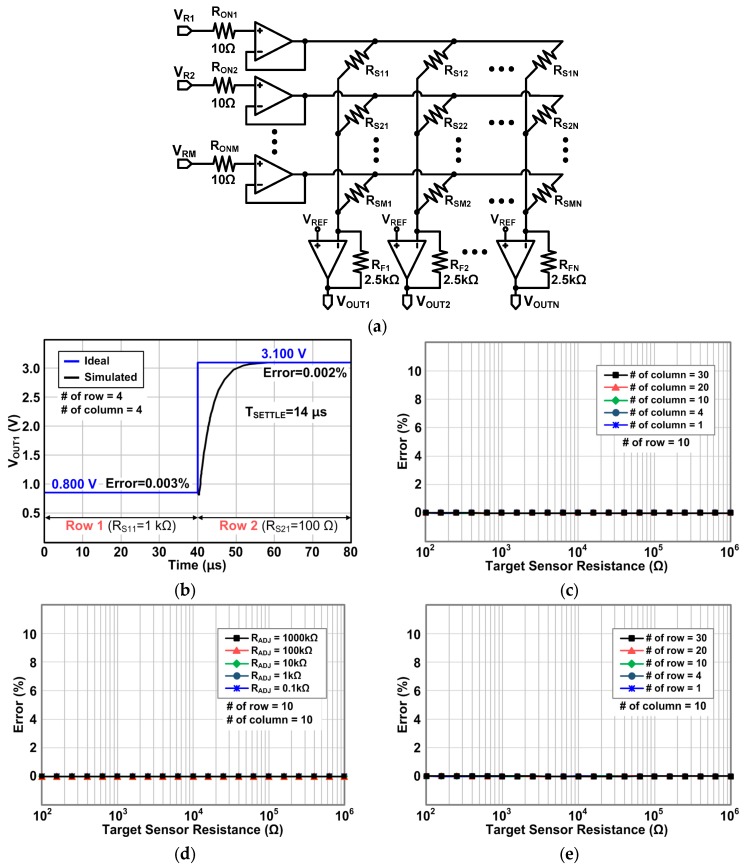
(**a**) Schematic for the simulations of the Type II row driver, (**b**) transient simulation results, (**c**) output errors as a function of the sensor resistance with different column numbers, (**d**) output errors as a function of the sensor resistance with different adjacent sensor resistance (R_ADJ_), and (**e**) output errors as a function of the sensor resistance with different row numbers.

Figure 11a shows the schematic for simulations of row driver Type II. As mentioned in Section 2, the selection switch is modeled as a resistor, R_ON_. All the simulation conditions are the same with the previous simulation with the Type I row driver. The transient simulation results of Type II are shown in Figure 11b. Unlike Type I, the Type II row driver creates a very small error even when the target sensor resistance is small (100 Ω). The simulated 99% settling time is 14 μs when a driven row is swiched from Row1 to Row 2.

Figure 11c shows the error of the Type II circuit as we sweep the target sensor resistance for a variety number of columns while fixing the number of rows at ten. As revealed in Equation (7) where the output voltage is only determined by a target sensor, not being affected by the other sensors in the same row, the simulation results show that the errors of the Type II circuit are very small: under 0.1%. In addition, as shown in Figure 11d, for different resitance of all the other sensor elements (R_ADJ_) from 100 Ω to 1000 kΩ, the Type II circuit still provides the small error under 0.1%. This is also evident in Equation (7) because there is no parameter from the other sensor elements, unlike Equations (2) and (6) for the Type I circuit. Therefore, we can conclude that the Type II row driver circuit can successfully resolve the inaccuracy problem, which is the main advantage of Type II. However, as described in Section 2, row driver Type II needs the same number of operational amplifiers as the rows; consequently, the complexity, fabrication cost, and power consumption are severely increased. Figure 11e shows the output voltage error for different numbers of rows with ten columns. For all different numbers of rows, the error remains very small under 0.1%.

**Figure 12:**
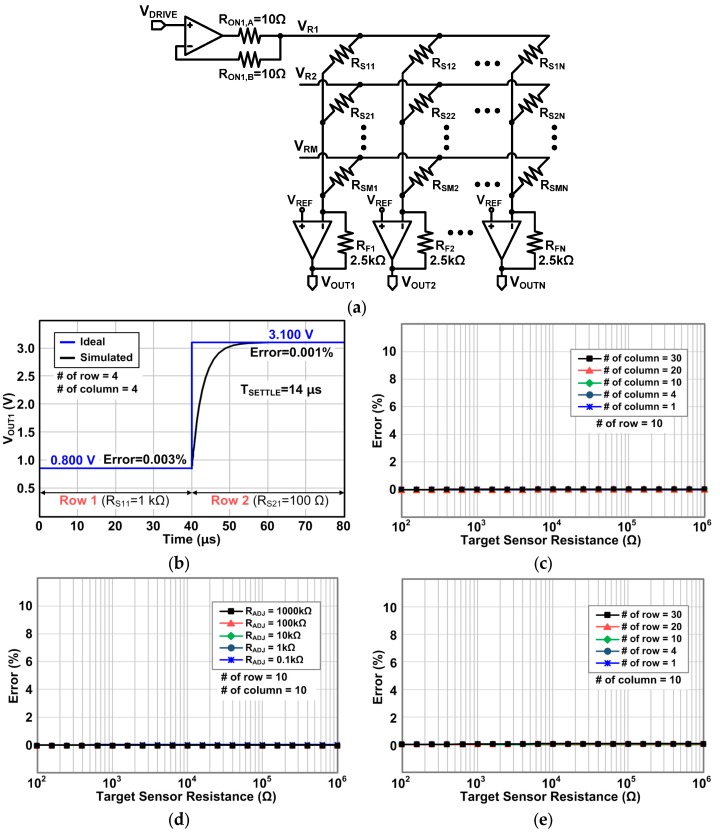
(**a**) Schematic for the simulations of the proposed row driver Type III; (**b**) transient simulation results; (**c**) output errors as a function of the sensor resistance with different column numbers; (**d**) output errors as a function of the sensor resistance with different adjacent sensor resistance(R_ADJ_); and (**e**) output errors as a function of the sensor resistance with different row numbers.

Figure 12a shows the schematic for simulations of the proposed Type III row driver. As described in Section 3, the selected row is connected to both the output and negative input of the operational amplifier, but the other row is disconnected (see Figure 8). All simulation conditions are the same with the previous simulations above. Figure 12b shows the transient simulation result of the proposed Type III row driver. Very similarly with Type II, the proposed Type III row driver also shows a very small error under 0.1% even when the target sensor resistance is small (100 Ω). The simulated 99% settling time is 14 μs which is equal to that of Type II row driver. This result agrees well with the expectation described in Section 3.

Figure 12c shows the error of the proposed Type III circuit as we sweep the target sensor resistance for a variety number of columns while fixing the number of rows at ten. The proposed Type III circuit also shows the low error under 0.1%. In addition, as shown in Figure 12d, for different resitance of all the other sensor elements (R_ADJ_) from 100 Ω to 1000 kΩ, the proposed Type III circuit provides the small error under 0.1% like the Type II circuit. Figure 12e shows the output voltage error for different numbers of rows with ten columns. For all different numbers of rows, the error remains very small, under 0.1% like the Type II circuit. From the results in Figure 12c–e, it is found that the Type III circuit can resolve the inaccuracy problem, like the Type II row driver, but with a single row amplifier.

**Figure 13:**
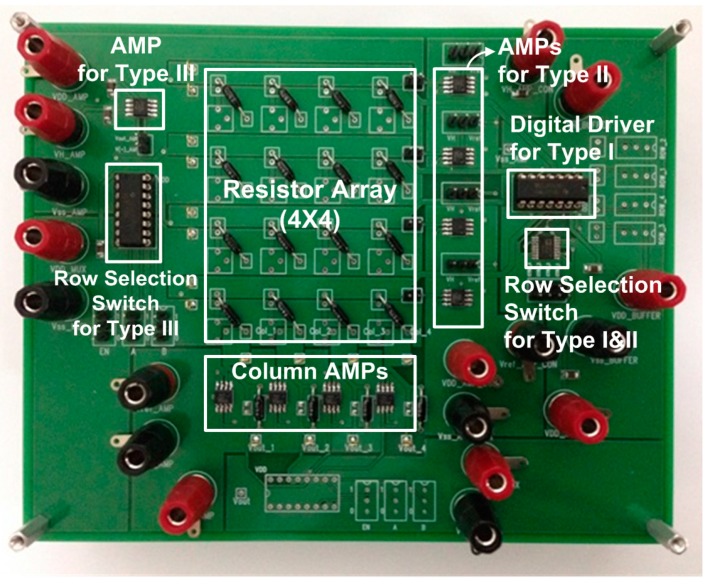
Test board with row drivers Type I, II, III, and 4 × 4 resistor array.

In addition to simulations, we also evaluated the characteristics of the previous row driver Type I, II, and the proposed Type III circuits, by establishing a 4 × 4 resistor array with those three types of row drivers on a printed circuit board (PCB) as shown in Figure 13. The high-precision amplifier, LMP7701 which is used for simulations above is also used for measurements. The high-accuracy resistors with the tolerance of 0.1% are used for the resistor array. A digital buffer (SN54AHCT126) with R_ON_ of about 15 Ω is used for row driver Type I [34]. The row-selection switch of the Type II row driver is realized with SPDT switches (ADG733) of which R_ON_ is typically 2.5 Ω [37]. The proposed Type III row driver uses an eight-channel multiplexer (CD4051B) of which R_ON_ is typically 125 Ω as a row selection switch [38]. As shown in the figure, the Type II row driver uses four operational amplifiers to drive four rows, but the proposed Type III uses only one operational amplifier.

**Figure 14:**
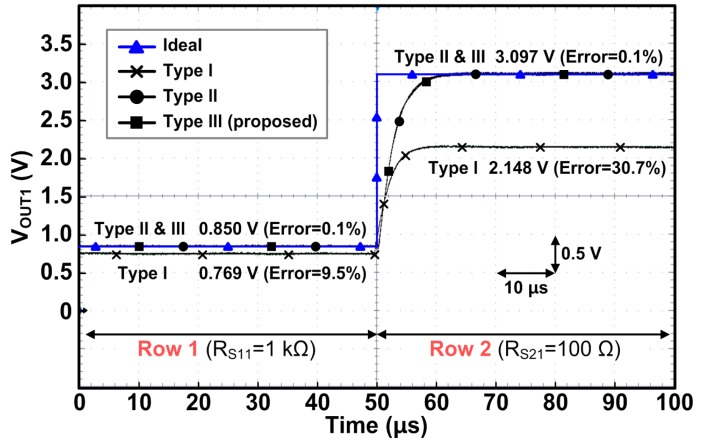
Measured waveforms of row drivers Type I, II, and the proposed Type III.

Figure 14 shows the measured waveforms of the previous Type I, II, and the proposed Type III row drivers with the 4 × 4 resistor array shown in Figure 13. Similar to the simulations above, when we select Row 1 we measure the value of R_S11_ by reading the output voltage at Column 1 (V_OUT1_). Since R_S11_ is assumed to be 1 kΩ in this experiment, V_OUT1_ should be 0.85 V, ideally, if there is no error. As shown in the figure, the error of the Type I row driver reaches 9.5%, but Type II and III row drivers show very small errors of 0.1%. Then, we select Row 2 and read the output voltage at Column 1 to find out R_S21_ (assumed to be 100 Ω here). As expected in Equation (6), the output error of Type I is further increased to 30.7%, but Type II and Type III row drivers still provide very small errors of 0.1%. Additionally, both Type II and Type III show the exactly identical transient response (rising time); thus, their dynamic characteristics are identical when the driven rows are switched.

**Figure 15:**
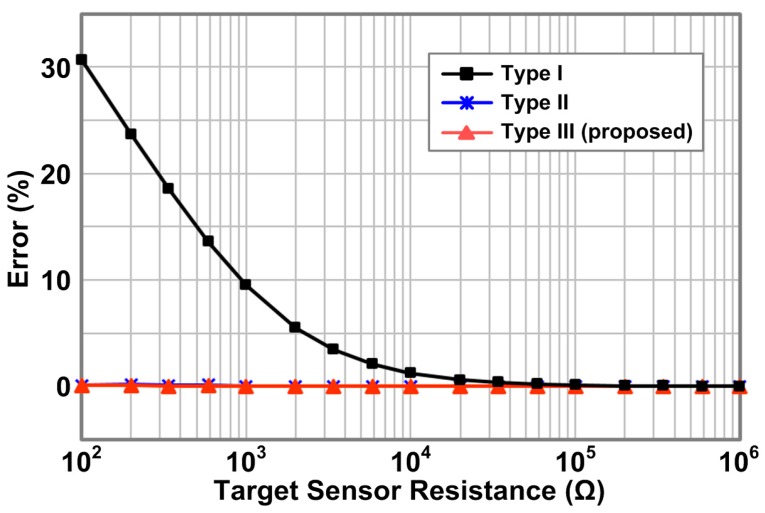
Measured output errors of three types of row driver circuits with 4 × 4 resistor array.

Figure 15 shows the measured output errors of the three types of row drivers with 4 × 4 resistor array as a function of the target sensor resistance while all the other resistors have 100 Ω. The target sensor resistance is swept from 100 Ω to 1 MΩ. As shown in the figure, row driver Type I shows severely large error over 30.7% as the target sensor resistance is decreased to a comparable value with all the other resistors (100 Ω), but the Type II and the proposed Type III row drivers provide small errors under 0.1%. From these measurement results, which agree very well with both the calculations and simulations in Section 2 and Section 3, we confirm that the Type I row driver suffers from the reading inaccuracy, but the Type II and the proposed Type III row drivers effectively solve the inaccuracy problem.

The performance comparison of the Type I, II, and the proposed Type III row driver circuits is summarized in Table 2. The proposed Type III row driver provides small error, but requires only one more operational amplifier than row driver Type I. Therefore, we can conclude that the proposed row driver Type III circuit is very efficient to remove the inaccuracy with little increase in the circuit complexity.

**Table 2:** Performance comparison of row drivers.

	Type I	Type II	Type III (Proposed)
Measured maximum error ^1^	30.7%	≤0.1%	≤0.1%
Number of operational amplifiers (for N × M array)	N	N + M	N + 1

^1^ Measurement conditions: 4 × 4 resistor array, target sensor resistance = 100 Ω, and all the other sensor resistance = 100 Ω.

## 5. Conclusions

In order to resolve the inaccuracy and high complexity problems found in the previous row drivers, we propose a new row driver circuit which uses only one operational amplifier to drive all rows of a sensor array. Although the proposed Type III row driver has only one operational amplifier to drive all the rows, it successfully removes the inaccuracy problem by using a proper feedback operation. With the reduced number of operational amplifiers, the proposed Type III circuit can achieve the low power, low cost, and low complexity which are strongly required for large sensor arrays. The PSPICE simulation results show that the proposed circuit provides a small error of under 0.1% error even with 30 columns of a sensor array. The measurement results from the circuit implementation show that the maximum error with a 4 × 4 resistor array is 0.1% which is remarkably reduced from 30.7% of the previous counterpart. From these improved performances, we can conclude that the proposed Type III row driver circuit is very well suited for resistive sensor array applications.
